# Impact of local left atrial wall thickness on the incidence of acute pulmonary vein reconnection after Ablation Index-guided atrial fibrillation ablation

**DOI:** 10.1016/j.ijcha.2020.100574

**Published:** 2020-07-03

**Authors:** Mark J. Mulder, Michiel J.B. Kemme, Amaya M.D. Hagen, Luuk H.G.A. Hopman, Peter M. van de Ven, Herbert A. Hauer, Giovanni J.M. Tahapary, Marco J.W. Götte, Albert C. van Rossum, Cornelis P. Allaart

**Affiliations:** a Department of Cardiology, Amsterdam UMC, Vrije Universiteit Amsterdam, Amsterdam Cardiovascular Sciences Amsterdam, the Netherlands; b Department of Epidemiology and Biostatistics, Amsterdam UMC, Vrije Universiteit Amsterdam, Amsterdam, the Netherlands; c Cardiology Centers of the Netherlands, Amsterdam, the Netherlands; d Department of Cardiology, North West Clinics, Alkmaar, the Netherlands

**Keywords:** Acute reconnection, Atrial fibrillation, Catheter ablation, Computed tomography, Pulmonary vein isolation, Wall thickness

## Abstract

**Background:**

Although Ablation Index (AI)-guided ablation facilitates creation of lesions of consistent depth, pulmonary vein (PV) reconnection is still commonly observed after AI-guided pulmonary vein isolation (PVI). The present study aimed to investigate the impact of local left atrial wall thickness on the incidence of acute PV reconnection after AI-guided atrial fibrillation (AF) ablation.

**Methods and results:**

Seventy patients (63% paroxysmal AF, 67% male, mean age 63 ± 8 years) who underwent preprocedural CT imaging and AI-guided AF ablation were studied. Occurrence of acute PV reconnection after initial PVI was assessed after a 30-minute waiting period. Ablation procedures were retrospectively analyzed and each ablation circle was subdivided into 8 segments. Minimum AI, force-time integral, contact force, ablation duration, power, impedance drop and maximum interlesion distance were determined for each segment. PV antrum wall thickness was assessed for each segment on reconstructed CT images based on patient-specific thresholds in Hounsfield Units. Acute reconnection occurred in 27/1120 segments (2%, 15 anterior/roof, 12 posterior/inferior) in 19/140 ablation circles (14%). Reconnected segments were characterized by a greater local atrial wall thickness, both in anterior/roof (1.87 ± 0.42 vs. 1.54 ± 0.42 mm; p < 0.01) and posterior/inferior (1.43 ± 0.20 vs. 1.16 ± 0.22 mm; p < 0.01) segments. Minimum AI, force-time integral, contact force, ablation duration, power, impedance drop and maximum interlesion distance were not associated with acute reconnection.

**Conclusions:**

Local atrial wall thickness is associated with acute pulmonary vein reconnection after AI-guided PVI. Individualized AI targets based on local wall thickness may be of use to create transmural ablation lesions and prevent PV reconnection after PVI.

## Highlights

• Ablation Index-guided ablation allows for ablation lesions of consistent depth.
• Ablation Index-guided ablation is limited by ignoring local wall thickness.
• Local atrial wall thickness is associated with acute pulmonary vein reconnection.
• Wall thickness adjusted Ablation Index targets may improve ablation outcomes.

## 1 Introduction

Pulmonary vein (PV) reconnection is mainly due to nontransmural ablation [1], and is considered a major determinant of atrial fibrillation (AF) recurrence after pulmonary vein isolation (PVI) [2]. Several technologies have been developed in an attempt to improve radiofrequency (RF) lesion transmurality and PVI durability, such as irrigated-tip ablation catheters and real-time contact force (CF)-sensing catheters [3], [4]. Recently, Ablation Index (AI; Carto3, Biosense Webster, Diamond Bar, CA) was introduced as a surrogate measure for ablation lesion quality and combines CF, ablation duration, and power into a weighted non-linear formula [5]. AI correlated strongly with ablation lesion depth in a canine model [6], and was shown to predict PV reconnection in patients undergoing CF-guided PVI [5], [7], [8]. Based on these findings, AI target values have been defined, ranging from 450 to 550 for anterior/roof segments and from 330 to 400 for posterior/inferior segments [9], [10], [11], [12], [13]. However, pathological and non-invasive imaging studies have demonstrated an important inter- and intra-patient variability of left atrial wall thickness [14], [15], [16], [17]. We hypothesized that the use of fixed AI targets could lead to non-transmural ablation lesions and subsequent PV reconnection in thicker atrial wall segments. The aim of the present study was therefore to investigate the impact of local left atrial wall thickness on the incidence of acute PV reconnection after AI-guided AF ablation.

## 2 Methods

### 2.1 Patient population

All patients undergoing AF ablation at Amsterdam UMC, location VU University medical center from 2017 to 2019 were approached for enrollment in a prospective registry with recording of clinical characteristics, imaging data, and procedural data. Patients who signed informed consent were enrolled in a research ethics board–approved registry. For the present study, patients who underwent cardiac computed tomography (CT) imaging prior to AI-guided AF ablation between December 2017 and September 2019 were investigated. Patients with a prior LA catheter ablation or LA surgery procedure were excluded from analysis. Paroxysmal AF was defined as AF that self-terminated or was cardioverted within 7 days of onset.

The principles outlined in the Declaration of Helsinki were followed. Written informed consent was obtained from all patients and the institutional medical ethics committee of the VU university medical center approved collection and management of data.

### 2.2 CT image acquisition and analysis

Patients underwent contrast-enhanced ECG-gated cardiac CT imaging using a 256-slice (Brilliance iCT, Philips Healthcare, Best, the Netherlands) scanner. If needed, beta blockers were administered to achieve a heart rate below 65 beats per minute. An intravenous bolus of iodinated contrast (Iobitridol or Iopromid) was administered at a rate of 6 mL/s followed by a flush of 45 to 50 mL saline. Scans were performed in a single breath hold and were initiated automatically by bolus tracking software after reaching a threshold of 150 Hounsfield Units (HU) in the descending aorta. Scan parameters were detector collimation of 128 × 0.625 mm, gantry rotation time of 270 ms, tube voltage between 100 and 140 kV and a tube current between 200 and 360 mAs, depending on body habitus. Non-overlapping images were reconstructed to 0.9 mm slice thickness with 0.4 mm slice increment, using iterative reconstruction with a 512 × 512 matrix at 75% of the R-R interval.

**Fig. 1:**
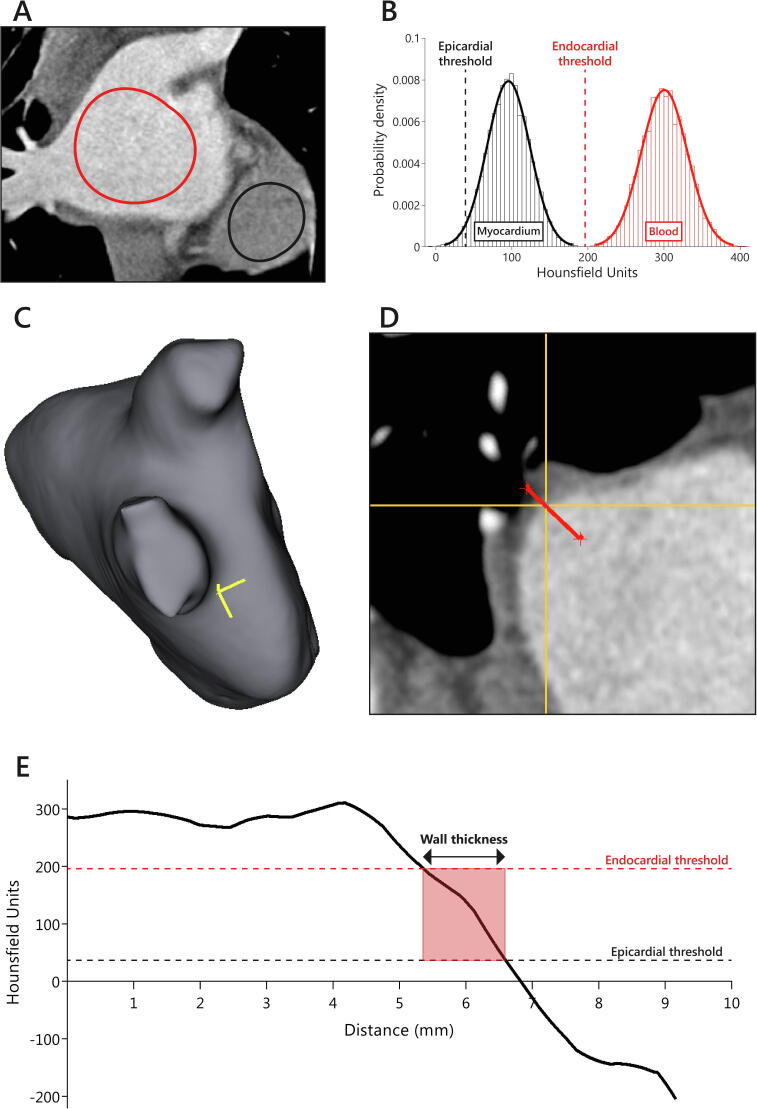
Example of wall thickness measurement. Panel A: Regions of interest were drawn inside the left atrium and ventricular apex. Panel B: Blood pool and myocardium intensity histograms were constructed from the regions of interest. The endocardial threshold value was calculated by averaging the means of the blood pool and myocardial image intensity. The epicardial threshold value was calculated as two standard deviations below the mean of the myocardial image intensity. Panel C: Locations for left atrial wall thickness measurement of the pulmonary vein (PV) antra were manually selected using a 3D segmentation of the left atrium. The yellow crosshair represents the location of wall thickness measurement on the anterior segment of the right inferior PV. Panel D: Multiplanar reformatted images were generated perpendicular to the atrial wall and the crosshair corresponds to the location chosen on the 3D segmentation (panel C). Subsequently, a line (in red) perpendicular to the atrial wall was drawn. Panel E: The intensity profile of the line perpendicular to the atrial wall was obtained. Atrial wall thickness was calculated using the patient-specific endocardial and epicardial thresholds. (For interpretation of the references to color in this figure legend, the reader is referred to the web version of this article.)

CT image analysis was performed blinded to ablation data using open-source software (3D Slicer 4.10.2, www.slicer.org). LA diameter was measured in the anteroposterior direction on axial non-reformatted images. LA wall thickness measurements were performed similarly as described previously [18], and as depicted in Fig. 1. Two separate circular regions of interest were drawn on coronal images inside the LA (blood pool) and inside the ventricular myocardium. Intensity histograms were constructed from the regions of interest to define mean and standard deviation HU for blood pool and myocardium. The endocardial threshold value was calculated by averaging the means of the blood pool and myocardial HU, whereas the epicardial threshold value was calculated as two standard deviations below the mean myocardial HU. A three-dimensional model of the LA blood pool was obtained by a region-growing segmentation algorithm, which was used to manually select locations for PV antral wall thickness measurements. Subsequently, multiplanar reformatted images perpendicular to the atrial wall were created and trilinear interpolation was used to create transformed images with 0.1 mm isotropic voxel dimensions. A line perpendicular to the atrial wall was drawn and its intensity profile was determined. Local atrial wall thickness was derived using the patient-specific endocardial and epicardial thresholds (Fig. 1).

The PV antrum was defined as the 10 mm section between the PV ostium and LA. A similar 16-segment model as for the ablation procedure data was used for PV antral wall thickness measurements. Measurements were repeated five times for each segment and the wall thickness was determined as the mean of measurements after discarding the highest and lowest value to exclude outliers. A total of 112 segments from 7 randomly selected patients underwent repeat review to assess intra- and inter-reader reliability.

### 2.3 Ablation procedure and analysis

Ablation procedures were performed under either conscious sedation, deep sedation, or general anesthesia. After administering a heparin bolus to achieve an activated clotting time of 300–400 s, transseptal puncture was performed using fluoroscopic guidance and continuous pressure monitoring. Fast anatomical mapping was performed using a 3D mapping system (Carto3, Biosense Webster) and a circular mapping catheter (Lasso, Biosense Webster) to reconstruct LA geometry.

Circumferential antral ablation was performed with an irrigated tip CF-sensing ablation catheter (Thermocool Smarttouch, Biosense Webster) to create a contiguous circle of lesions enclosing ipsilateral PVs. Automated real-time lesion tagging software (VisiTag™, Biosense Webster) was used with fixed settings: stability maximum distance change 3 mm, minimum time 8 s and force over time set at 30% with a minimum force of 5 g. Catheter stability was assessed during RF applications, with a target contact force of 5–40 g. Ablation lesions were created in a point-by-point fashion, aiming for a maximum center-to-center interlesion distance of 6 mm and minimum AI values of 500 for anterior/roof segments and 380 for posterior/inferior segments. Power settings were 40 W for anterior/roof segments and 30/35 W for posterior/inferior segments. Touch-up RF applications were performed if needed to achieve PVI. Durability of PVI was assessed after a waiting period of 30 min, without adenosine testing. If acute reconnection occurred, the site of reconnection was noted and further ablation was performed until re-isolation was achieved. Additional cavotricuspid isthmus ablation was performed in patients with documented typical atrial flutter.

**Fig. 2:**
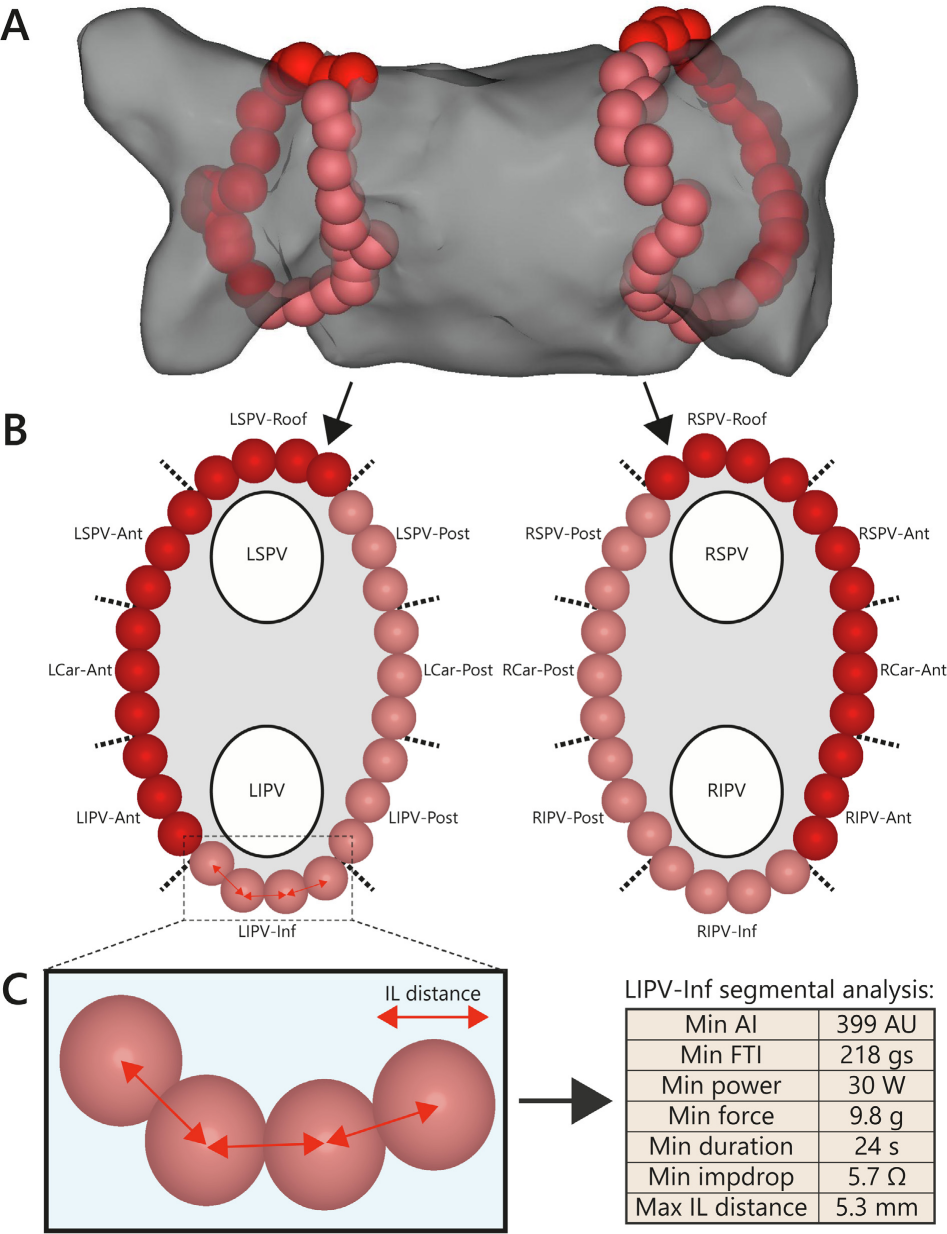
Ablation procedure analysis. Panel A: Posteroanterior projection of a typical circumferential antral ablation approach. Ablation tags are automatically color coded based on Ablation Index (AI) values. Pink ablation tags represent AI values 380 to 499, whereas red tags represent AI values > 500. Panel B: Ablation tags were classified according to a 16-segment model for segmental analysis. Ablation tags belonging to additional ablation applications to treat acute pulmonary vein reconnection were excluded from analysis. Panel C: Minimum AI value, force–time integral (FTI), contact force (CF), ablation duration, power, impedance drop (impdrop) and maximum interlesion (IL) distance were determined for each segment. Ant = anterior, AU = arbitrary units, Inf = inferior, LCar = left carina, LIPV = left inferior pulmonary vein, LSPV = left superior pulmonary vein, Post = posterior, RCar = right carina, RIPV = right inferior pulmonary vein, RSPV = right superior pulmonary vein. (For interpretation of the references to color in this figure legend, the reader is referred to the web version of this article.)

All procedures were analyzed offline on a dedicated Carto3 workstation. Each ablation circle was divided into 8 segments and minimum and mean AI, force–time integral (FTI), CF, ablation duration, power, and impedance drop were noted for each segment (Fig. 2). The Carto3 built-in “distance measurement tool” was used to determine maximum and mean center-to-center interlesion distance for each segment. Impedance drop was defined as the maximum difference between pre-ablation impedance and recorded impedance values during ablation. The circumference of the ablation circle was measured through all ablation tags using the Carto3 built-in “design line tool”. Ablation lesions targeting sites of acute PV reconnection were excluded from segmental analysis.

### 2.4 Statistical analysis

All analyses were performed in SPSS version 22 (IBM Corporation, Armonk, NY, USA). The level of statistical significance was set at a two-sided P value < 0.05. Normality of continuous data was assessed by inspection of histograms and Q-Q plots. Continuous variables are expressed as mean ± standard deviation or as median [interquartile range], where appropriate. Categorical variables are presented as frequency (percentage) and were compared using the chi-square test. Incidence of acute reconnection was compared between anterior/roof and posterior/inferior segments and between left-sided and right-sided segments using Generalized Estimating Equations for a binary outcome with a logit link. To account for within-subject correlation an exchangeable correlation structure was used together with a robust estimator for the covariance matrix. Comparisons of ablation parameters and local wall thickness were made separately for anterior/roof segments and posterior/inferior segments and were performed using linear mixed models to account for within-patient correlations. Receiver operating characteristics (ROC) curve analysis was performed to calculate the ability of minimum FTI, minimum AI, and minimum AI adjusted to local wall thickness (AI/wall thickness) to discriminate between overall segments with and without acute PV reconnection. Intra- and inter-observer variability of LA wall thickness measurements was quantified by means of intraclass correlation coefficients for absolute agreement based using a two-way random model.

## 3 Results

Seventy symptomatic AF patients who underwent cardiac CT imaging prior to AI-guided ablation were studied. The mean age of the patients was 63 ± 8 years, 47 (67%) were male and 43 (63%) had paroxysmal AF. Median time from diagnosis of AF to ablation procedure was 38 [16–94] months. Patient characteristics are summarized in Table 1. Conscious sedation was used in 16 procedures (23%), deep sedation was used in 52 procedures (74%), and 2 procedures (3%) were performed under general anesthesia. Cavotricuspid isthmus ablation for typical atrial flutter was performed in 15 patients (21%). No major complications were observed.

**Table 1:** Patient characteristics.

Characteristic	AI-guided PVI (n = 70)
Age (years)	63 ± 8
Male	47 (67%)
Paroxysmal AF	44 (63%)
Body length (cm)	179 ± 10
Weight (kg)	86 ± 14
Body mass index (kg/m^2^)	26.9 ± 3.6
Body surface area (m^2^)	2.1 ± 0.2
Congestive heart failure	7 (10%)
Hypertension	23 (33%)
Diabetes mellitus	3 (4%)
History of stroke/TIA	6 (9%)
Coronary artery disease	9 (13%)
CHA2DS2-VASC score	1.6 ± 1.3
AF duration (months)	38 [16–94]
Previous anti-arrhythmic drug use	67 (96%)
Left atrial anteroposterior diameter (mm)	40 ± 7
Blood pool intensity mean (HU)	361 ± 87
Blood pool intensity SD (HU)	52 ± 20
Myocardium intensity mean (HU)	74 ± 20
Myocardium intensity SD (HU)	38 ± 12
Epicardial threshold (HU)	218 ± 47
Endocardial threshold (HU)	−2 ± 28
Time from first to last RF application (min.)	86 ± 27
Total RF duration (min.)	35 ± 7
Total fluoroscopy duration (min.)	8 ± 4
Left ablation circle circumference (mm)	119.8 ± 23.1
Right ablation circle circumference (mm)	125.7 ± 23.7

All values are mean ± SD or median [IQR] for continuous variables and number (%) for categorical variables.

AF = atrial fibrillation, AI = Ablation Index, HU = Hounsfield units, PVI = pulmonary vein isolation, RF = radiofrequency, TIA = transient ischemic attack.

### 3.1 Ablation procedure analysis

**Fig. 3:**
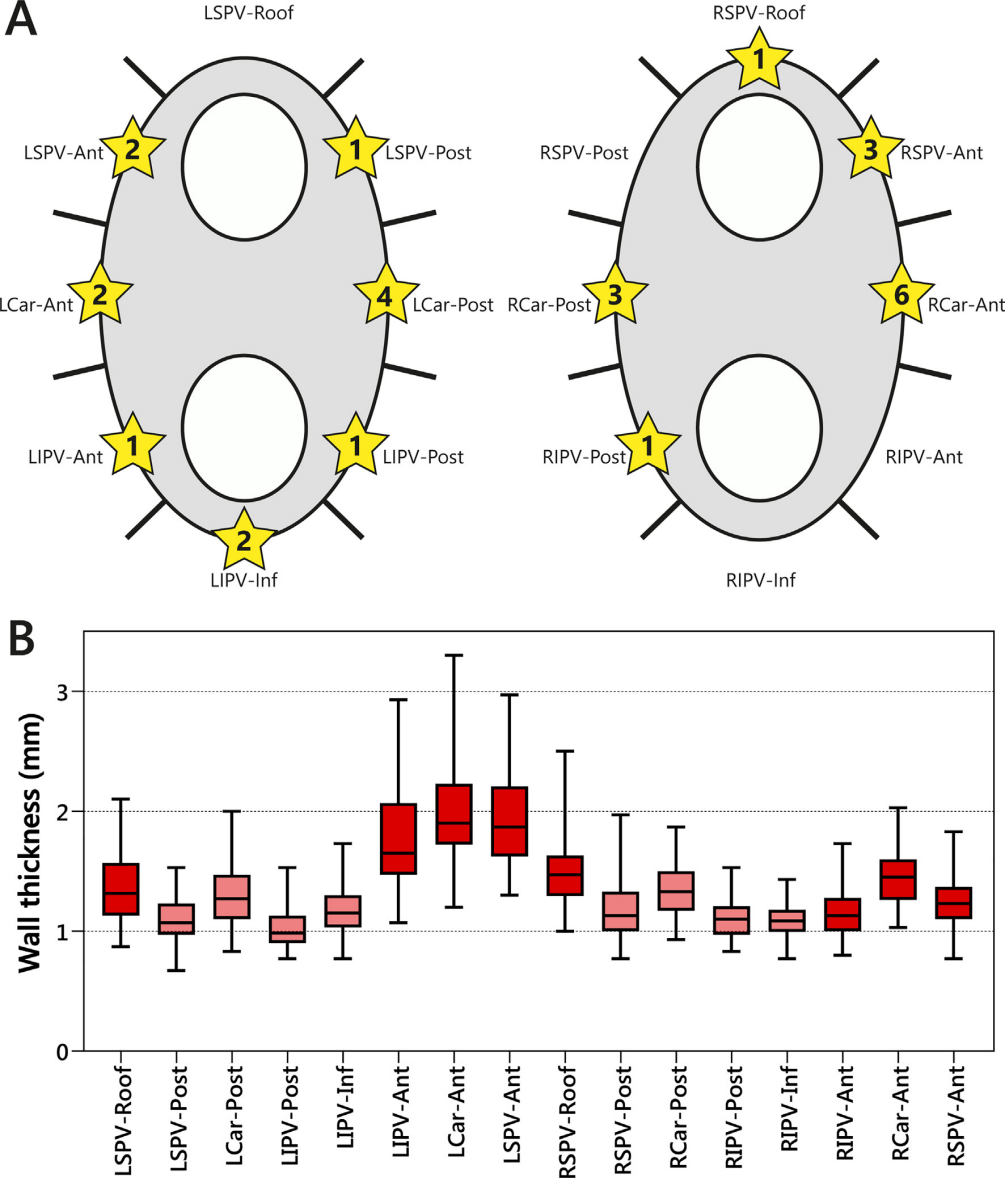
Acute reconnection and wall thickness per segment. Panel A: Sites of acute pulmonary vein reconnection were defined according to a 16-segment model. Numbers inside the stars indicate the total number of acute reconnections per segment. Panel B: Box plots showing local left atrial wall thickness per segment. Anterior/superior segments and posterior/inferior segments are displayed by red and pink colors, respectively. Ant = anterior, Inf = inferior, LCar = left carina, LIPV = left inferior pulmonary vein, LSPV = left superior pulmonary vein, Post = posterior, RCar = right carina, RIPV = right inferior pulmonary vein, RSPV = right superior pulmonary vein. (For interpretation of the references to color in this figure legend, the reader is referred to the web version of this article.)

A total of 4848 automatically generated ablation tags were analyzed and subdivided into a total of 1120 segments. All 280 PVs were successfully isolated with 88% of PVs isolated after first-pass. After a 30-minute waiting period, acute reconnection was observed in 27/1120 segments (2%) in 19/140 ablation circles (14%). The incidence of acute reconnection was similar for anterior/roof and posterior/inferior segments (15/560 vs. 12/560 segments; p = 0.54), as well as for left-sided and right-sided segments (13/560 vs. 14/560 segments; p = 0.87). The distribution of reconnected segments is depicted in Fig. 3A.

An overview of ablation parameters per segment is presented in Supplemental Table 2. Compared with posterior/inferior segments, anterior/roof segments were characterized by a greater minimum AI (508 [504–513] vs. 394 [387–405] AU; p < 0.01), minimum FTI (289 ± 61 vs. 222 ± 62 gs; p < 0.01), minimum ablation duration (22.5 ± 5.5 vs. 18.1 ± 4.9 s; p < 0.01), minimum power (41 [40–41] vs. 31 [30–31] W; p < 0.01), minimum impedance drop (7.4 ± 4.2 vs. 5.4 ± 3.4 Ω; p < 0.01), and maximum interlesion distance (5.0 [4.4–5.6] vs. 4.8 [4.3–5.3] mm; p < 0.01). Minimum CF was similar for anterior/roof and posterior/inferior segments (9.5 [7.8–11.8] vs. 9.1 [7.5–11.5] g; p = 0.16).

### 3.2 Atrial wall thickness measurements

Regional differences in atrial wall thickness were noted, as shown in Fig. 3B. Anterior/roof segments were thicker than posterior/inferior segments (1.55 ± 0.43 vs. 1.16 ± 0.23 mm; p < 0.01) and left-sided segments were thicker than right-sided segments (1.45 ± 0.46 vs. 1.26 ± 0.27 mm; p < 0.01). Greater wall thickness was observed in carina segments compared to non-carina segments (1.51 ± 0.39 vs. 1.30 ± 0.38 mm; p < 0.01).

The intraclass correlation coefficient for intra-reader variability of atrial wall thickness measurements was 0.91 (95% confidence interval: 0.87–0.94). The intraclass correlation coefficient for inter-reader variability of atrial wall thickness measurements was 0.87 (95% confidence interval: 0.81–0.91).

### 3.3 Segments with and without acute reconnection

**Fig. 4:**
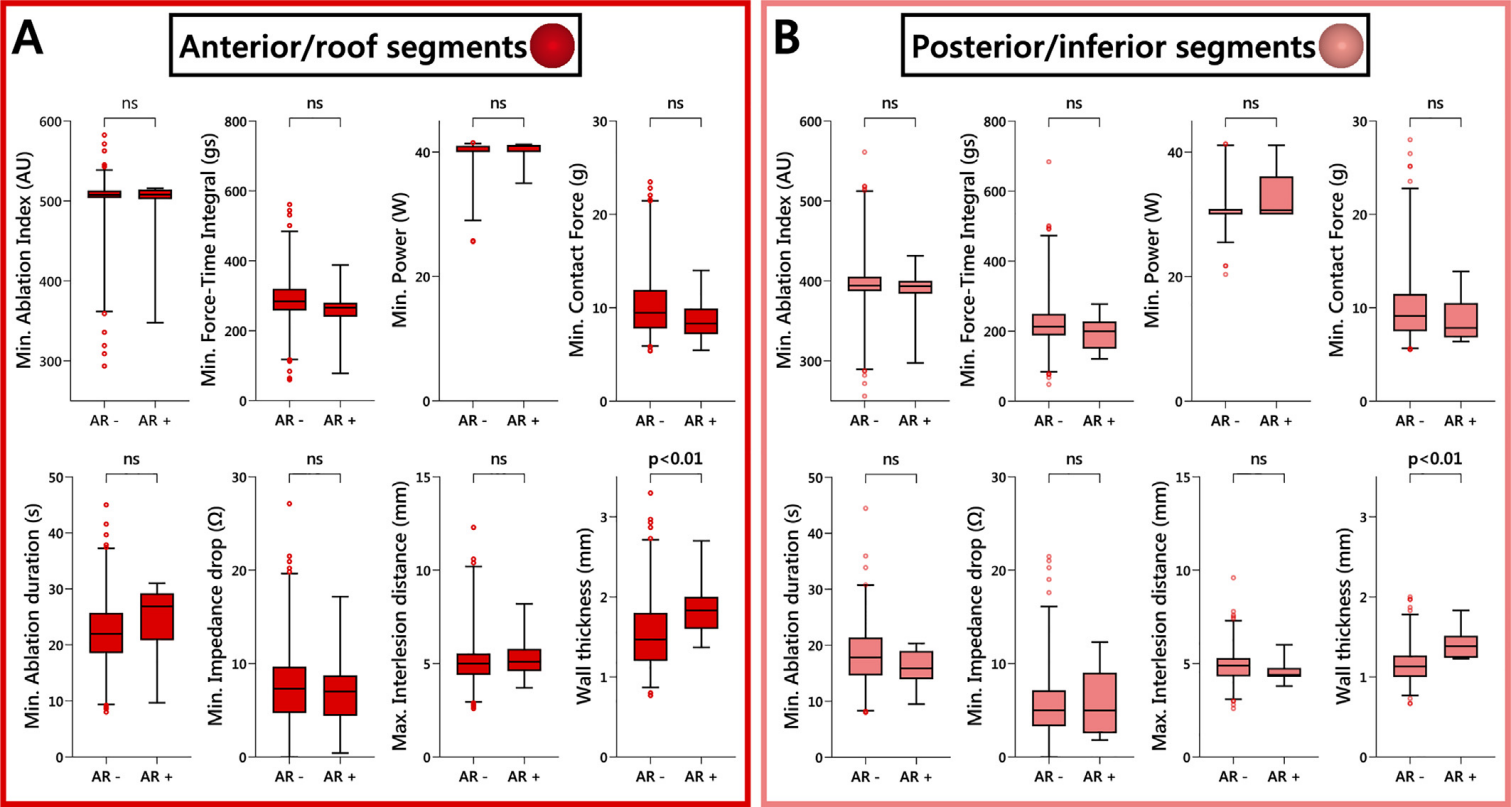
Comparison of reconnected and non-reconnected segments. Box plots showing difference in minimum Ablation Index value, force-time integral, contact force, ablation duration, power, impedance drop, maximum interlesion distance, and wall thickness between segments with acute reconnection (AR +) and segments without acute reconnection (AR −) for anterior/roof segments (panel A) and posterior/inferior segments (panel B). ns = not significant.

Reconnected segments were characterized by a greater local atrial wall thickness, both in anterior/roof (1.87 ± 0.42 vs. 1.54 ± 0.42 mm; p < 0.01) and posterior/inferior (1.43 ± 0.20 vs. 1.16 ± 0.22 mm; p < 0.01) segments (Fig. 4). A segmental comparison of local atrial wall thickness between reconnected and non-reconnected segments is presented in Supplemental Table 2. Minimum and mean AI, FTI, CF, ablation duration, power, and impedance drop were not associated with acute reconnection of either anterior/roof or posterior/inferior segments. Similarly, maximum and mean interlesion distance were both comparable between anterior/roof segments with and without acute reconnection and posterior/inferior segments with and without acute reconnection.

ROC curve analysis of all segments (Supplemental Fig. 1) revealed that minimum FTI (AUC = 0.60; p = 0.07) and unadjusted minimum AI (AUC = 0.52; p = 0.69) did not discriminate between segments with and without acute reconnection, but minimum AI adjusted to local wall thickness did (AUC = 0.78; p < 0.01). For minimum AI adjusted to local wall thickness, a cut-off value of 377.4 had a negative predictive value of 100% (sensitivity 36.3%, specificity 100%, positive predictive value 3.9%).

## 4 Discussion

This is the first study to analyze the impact of local wall thickness on the occurrence of acute PV reconnection in patients undergoing AI-guided AF ablation. The main findings are as follows: (1) marked regional variability in PV antral wall thickness was noted; (2) local wall thickness was associated with occurrence of acute PV reconnection, both in anterior/roof segments and posterior/inferior segments and (3) commonly used metrics of ablation lesion quality such as AI, FTI, and impedance drop did not correlate with PV reconnection, whereas AI adjusted to wall thickness had predictive value for PV reconnection.

### 4.1 Ablation index-guided ablation

Recovery of electrical conduction after initial PVI occurs in a significant proportion of patients undergoing CF-guided ablation [19], [20]. Guidance of ablation by CF or FTI is limited by not taking into account the effects of ablation power on ablation lesion size. AI combines CF, ablation duration, and power into a single value that correlated strongly with lesion depth in a canine study and was associated with impedance drop during AF ablation [6]. Das et al. [5] were first to demonstrate predictive value of AI for acute PV reconnection and late PV reconnection at repeat electrophysiology procedure. In addition, the authors found that minimum AI to prevent PV reconnection was higher for anterior/roof segments compared to posterior/inferior segments. El Haddad et al. [7] showed that PV reconnection could be due to insufficient lesion depth as well as insufficient lesion contiguity, i.e. low minimum AI or large interlesion distance.

Recently, several studies compared the effectiveness of AI-guided ablation and conventional CF-guided ablation for AF. Despite using varying AI targets, ranging from 450 to 550 for anterior/roof segments and from 350 to 400 for posterior/inferior segments, reduced AF recurrence rates with AI-guided ablation were consistently observed.[10], [11], [12] Moreover, AI-guided AF ablation was associated with improved procedural durability of PVI, compared to CF-guided ablation [10], [11], [12]. Nevertheless, acute reconnection was observed in 1–14% of PVs after AI-guided ablation [9], [10], [11], [12], and at least 1 PV was reconnected in 22–38% of patients undergoing a repeat procedure during follow-up [9], [21]. In contrast to PV reconnections after CF-guided ablation, PV reconnections after AI-guided ablation were not attributable to lesions with low AI [9]. The present study confirmed this finding, showing that acute reconnections after ablation guided by AI targets could neither be explained by low AI or other markers of lesion quality, nor by large interlesion distance.

### 4.2 Importance of local atrial wall thickness

The different target AI values for anterior/roof and posterior/inferior segments were postulated to be caused by regional differences in atrial wall thickness [5], [7]. In this study, anterior/roof segments were indeed thicker than posterior/inferior segments. However, fixed AI targets for anterior/roof and posterior/inferior segments do not fully reflect the considerable inter- and intra-patient variability in LA wall thickness. We found that left anterior segments at the left lateral ridge were thickest and that carina segments were thicker than non-carina segments. Of note, the left lateral ridge and carina region are generally considered preferential sites for PV reconnection [22], which was also observed in the present study.

Previous research showed that PV reconnection after PVI occurred more frequently in thicker segments [17], [23], [24]. In these studies, ablation was guided by either ablation duration or electrogram attenuation, and no specific AI/FTI thresholds were targeted. Comparing our results with these data, the proportion of reconnected segments was lower in our cohort (2% vs. 4–20%), conceivably due to the use of strict criteria for lesion depth and contiguity. Nevertheless, our findings add to the existing evidence demonstrating more frequent PV reconnection in thicker segments, both in anterior/roof and posterior/inferior segments. Moreover, we found that minimum AI adjusted to wall thickness was associated with acute PV reconnection, whereas minimum FTI and unadjusted minimum AI could not discriminate between segments with and without reconnection.

A gold standard for atrial wall thickness measurements is currently not available. The tissue fixing process may impact measurements in histological studies, whereas variable image quality and limited spatial resolution may hamper measurements in imaging studies [25]. Owing to its high spatial resolution, CT imaging is considered the optimal non-invasive modality for assessing atrial wall thickness. Cardiac magnetic resonance (CMR) imaging has also been used in previous studies to assess atrial wall thickness [25]. CMR can simultaneously provide valuable information on myocardial tissue characteristics and scar, which may affect ablation lesion formation [26]. However, CMR-based wall thickness measurement methods may be limited by insufficient spatial resolution. Previous CT imaging studies on atrial wall thickness have mostly relied on manual ruler-based measurements with visual assessment of endocardial and epicardial myocardial borders [17], [23], [24]. Inoue et al. [18] developed a wall thickness measurement technique based on patient-specific endocardial and epicardial thresholds, which was also adopted in this study. Although pathologic validation and comparative studies with other measurement methods are currently lacking, the semi-automatic process of defining endocardial and epicardial borders in this method may increase accuracy and reproducibility. In the present study good to excellent intra- and interobserver reliability of wall thickness measurements was observed.

Bishop et al. [16] demonstrated the feasibility of generating three-dimensional models of the left atrium from CT imaging data with local wall thickness projected on the model surface. Integration of such three-dimensional models into an electroanatomic mapping system has the potential to assist AF ablation. To date, no prospective ablation studies have been performed investigating the use of individualized lesion depth targets based on local wall thickness. Raising AI targets in thicker segments may likely improve lesion transmurality and could consequently improve durability of PVI. Conversely, lowering AI targets in thinner segments may reduce procedure duration and improve safety by avoiding unnecessary RF application. It should be noted that as a result of the low incidence of PV reconnection, the positive predictive value of AI/wall thickness was low. Therefore, use of the AI/wall thickness cut-off cannot fully avoid unnecessary ablation.

### 4.3 Limitations

Some limitations of this study need to be considered. First, the present non-randomized observational study is limited by sample size and relatively low PV reconnection rate. Second, durability of PVI was not assessed after the initial procedure. Future studies are required to assess the impact of local wall thickness on late PV reconnection and AF recurrence following ablation. Third, adenosine (triphosphate) administration to reveal dormant PV conduction may increase the number of reconnected PVs and was not performed in this study. In previous cohorts of AF patients who underwent PVI without the use of AI targets, adenosine-induced dormant conduction was associated with a thickened atrial wall [17], [24]. Nevertheless, it remains unclear whether these results can be extrapolated to AI-guided AF ablation. Finally, in this study we did not investigate all known factors that can influence transmural ablation lesion creation and can thus impact the PV reconnection rate. Catheter stability during ablation was not studied, and may be further improved with the use of high-frequency jet ventilation during general anesthesia [27]. Moreover, oblique or parallel catheter orientation during ablation and differences in myocardial tissue characteristics may hamper the reliability of AI to accurately reflect ablation lesion size [28], [29].

## 5 Conclusion

Local atrial wall thickness is associated with acute PV reconnection after AF ablation guided by AI. Further studies are warranted to investigate whether individualized AI targets based on local wall thickness can improve transmural lesion creation and prevent PV reconnection after PVI.

## Sources of funding

None.

## Declaration of Competing Interest

M.K. and C.A. have received institutional research and training grants from Biosense Webster, Inc.
