# Supplementary material for: Impact of local left atrial wall thickness on the incidence of acute pulmonary vein reconnection after Ablation Index-guided atrial fibrillation ablation
Source: Int J Cardiol Heart Vasc. 2020 Jul 3;29:100574. doi: 10.1016/j.ijcha.2020.100574 (PMC7334811; doi:10.1016/j.ijcha.2020.100574)
Supplement: Supplementary data 1 [file mmc1.docx]

**SUPPLEMENTAL MATERIAL**

**Impact of local left atrial wall thickness on the incidence of acute pulmonary vein reconnection after Ablation Index-guided atrial fibrillation ablation**

Mark J. Mulder, M.D.; Michiel J.B. Kemme, M.D, Ph.D.; Amaya M.D. Hagen, M.D. ; Luuk H.G.A. Hopman, M.Sc; Peter M. van de Ven, Ph.D., M.Sc., M.A.; Herbert A. Hauer, M.D., Ph.D.; Giovanni J.M. Tahapary, M.D.; Marco J.W. Götte, M.D., Ph.D.; Albert C. van Rossum, M.D., Ph.D.; Cornelis P. Allaart, M.D., Ph.D.

**Supplemental figure**

Figure 1. ROC curve analysis for the prediction of acute pulmonary vein reconnection

**Supplemental tables**

Table 1. Ablation parameters per segment

Table 2. Segmental comparison of local atrial wall thickness between reconnected and non-reconnected segments

**
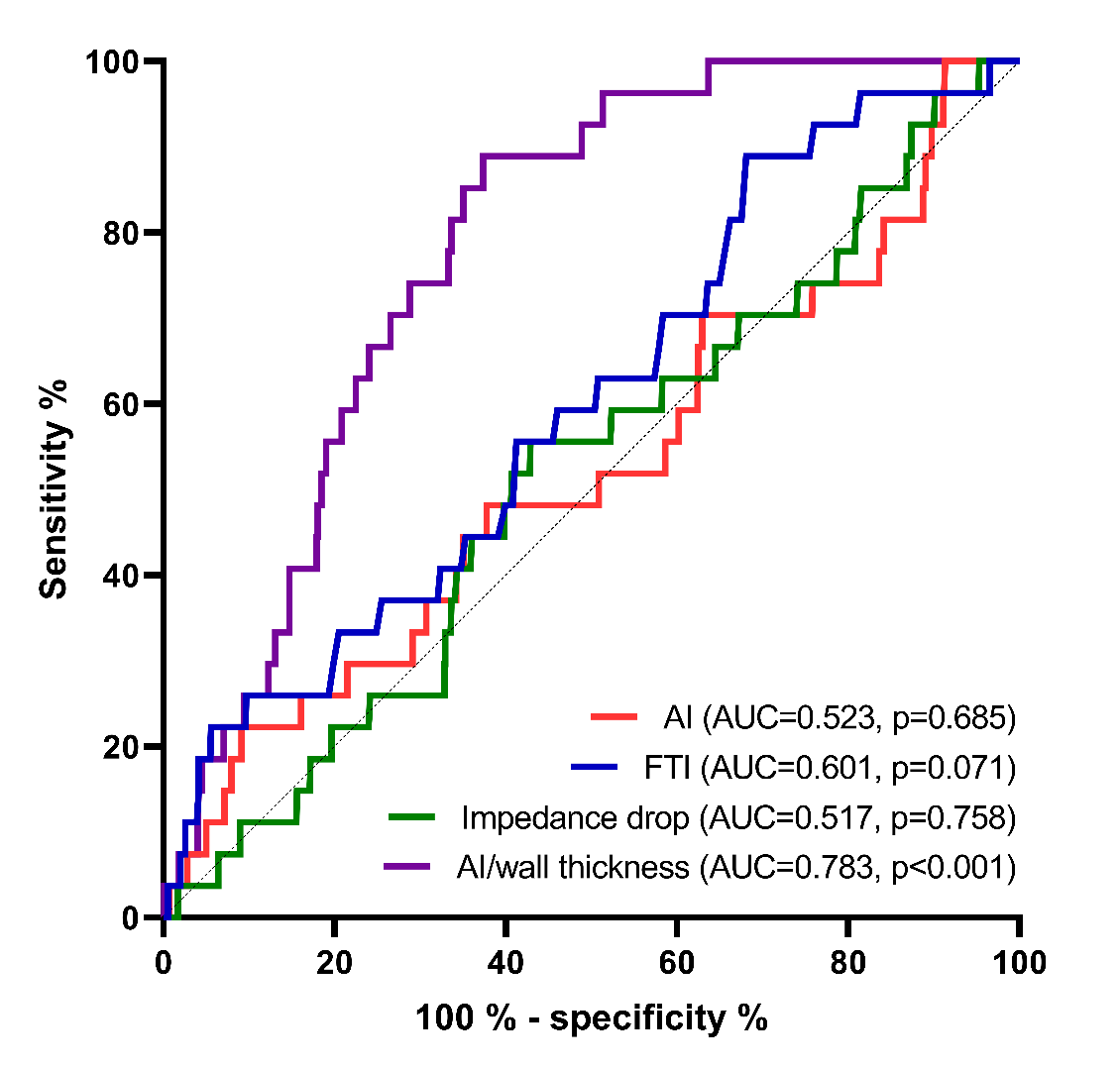
**

**Supplemental figure 1.**

Receiver operating characteristic (ROC) curve analysis for the prediction of acute pulmonary vein reconnection. AI = Ablation Index, AUC = area under curve, FTI = force-time integral

**Supplemental table 1. Ablation parameters per segment**

| **Segment** | **Min. AI (AU)** | **Min. FTI (gs)** | **Min. power (W)** | **Min. CF (g)** | **Min. duration (s)** | **Min. impdrop (Ω)** | **Max. IL distance (mm)** |
| --- | --- | --- | --- | --- | --- | --- | --- |
| **LSPV-Roof** | 507 [504-511] | 291 ± 49 | 40 [35-41] | 10.3 [8.3-12.4] | 21.2 ± 5.4 | 8.6 ± 4.5 | 5.1 [4.6-5.6] |
| **LSPV-Post** | 397 [389-408] | 235 ± 62 | 30 [30-31] | 9.4 [7.9-11.7] | 16.4 ± 3.6 | 7.2 ± 4.0 | 4.7 [4.2-5.2] |
| **LCar-Post** | 394 [388-403] | 225 ± 53 | 30 [30-31] | 10.4 [8.4-12.7] | 16.9 ± 4.4 | 6.4 ± 3.4 | 4.6 [4.2-5.2] |
| **LIPV-Post** | 393 [386-400] | 208 ± 53 | 30 [30-31] | 8.6 [6.8-11.4] | 17.9 ± 5.0 | 5.6 ± 3.3 | 4.8 [4.3-5.2] |
| **LIPV-Inf** | 396 [387-419] | 214 ± 58 | 30 [30-36] | 7.9 [6.9-9.7] | 19.7 ± 5.8 | 6.3 ± 3.6 | 5.0 [4.4-5.4] |
| **LIPV-Ant** | 507 [501-510] | 274 ± 65 | 41 [40-41] | 9.0 [7.2-11.2] | 23.1 ± 6.3 | 7.3 ± 3.8 | 5.0 [4.4-5.5] |
| **LCar-Ant** | 507 [505-513] | 264 ± 50 | 41 [40-41] | 8.2 [7.4-10.2] | 22.8 ± 6.4 | 7.6 ± 5.1 | 5.5 [4.9-6.4] |
| **LSPV-Ant** | 507 [505-512] | 279 ± 45 | 41 [40-41] | 8.9 [7.5-11.3] | 21.9 ± 5.0 | 6.7 ± 3.7 | 5.2 [4.5-5.8] |
| **RSPV-Roof** | 507 [496-512] | 290 ± 66 | 41 [38-41] | 9.0 [7.5-11.7] | 21.1 ± 5.9 | 5.9 ± 3.0 | 5.2 [4.6-5.6] |
| **RSPV-Post** | 396 [387-413] | 227 ± 78 | 30 [30-31] | 9.3 [8.0-10.5] | 17.6 ± 5.4 | 4.1 ± 2.7 | 5.4 [5.0-5.7] |
| **RCar-Post** | 393 [387-403] | 228 ± 69 | 30 [30-31] | 9.7 [8.2-11.9] | 18.5 ± 6.0 | 4.4 ± 2.5 | 4.8 [4.3-5.3] |
| **RIPV-Post** | 392 [387-401] | 220 ± 47 | 30 [30-31] | 8.7 [7.4-12.1] | 19.3 ± 3.8 | 4.0 ± 2.7 | 4.7 [4.2-5.1] |
| **RIPV-Inf** | 394 [386-401] | 218 ± 66 | 30 [30-31] | 8.6 [7.4-11.4] | 18.0 ± 3.6 | 5.5 ± 3.1 | 4.8 [4.4-5.2] |
| **RIPV-Ant** | 505 [498-514] | 313 ± 57 | 40 [32-41] | 11.5 [9.2-14.0] | 23.0 ± 4.1 | 8.7 ± 4.3 | 4.8 [4.2-5.3] |
| **RCar-Ant** | 509 [505-515] | 303 ± 65 | 41 [40-41] | 10.0 [8.3-12.1] | 22.9 ± 4.8 | 7.9 ± 4.2 | 4.8 [4.3-5.2] |
| **RSPV-Ant** | 508 [504-516] | 300 ± 76 | 41 [40-41] | 9.7 [8.1-11.9] | 23.7 ± 5.5 | 6.7 ± 4.0 | 5.0 [4.3-5.5] |

All values are mean ± SD or median [IQR].
AI = Ablation Index, Ant = anterior, AU = arbitrary units, CF = contact force, FTI = force-time integral, IL = interlesion, Impdrop = impedance drop, Inf = inferior, LCar = left carina, LIPV = left inferior pulmonary vein, LSPV = left superior pulmonary vein, Post = posterior, RCar = right carina, RIPV = right inferior pulmonary vein, RSPV = right superior pulmonary vein.

**Supplemental table 2. Segmental comparison of local atrial wall thickness between reconnected and non-reconnected segments**

| **Segment** | **Number of reconnected segments** | **Non-reconnected segments (mm)** | **Reconnected segments (mm)** | **p value** |
| --- | --- | --- | --- | --- |
| **LSPV-Roof** | 0 | 1.32 [1.13-1.57] | NA | NA |
| **LSPV-Post** | 1 | 1.07 [0.97-1.23] | 1.53 [1.53-1.53] | 0.091 |
| **LCar-Post** | 4 | 1.27 [1.10-1.47] | 1.37 [1.25-1.65] | 0.244 |
| **LIPV-Post** | 1 | 0.97 [0.90-1.13] | 1.23 [1.23-1.23] | 0.164 |
| **LIPV-Inf** | 2 | 1.13 [1.03-1.30] | 1.28 [1.23-1.33] | 0.273 |
| **LIPV-Ant** | 1 | 1.63 [1.47-2.03] | 2.17 [2.17-2.17] | 0.265 |
| **LCar-Ant** | 2 | 1.90 [1.72-2.22] | 2.23 [1.93-2.53] | 0.251 |
| **LSPV-Ant** | 2 | 1.87 [1.62-2.20] | 2.35 [2.00-2.70] | 0.148 |
| **RSPV-Roof** | 1 | 1.47 [1.30-1.63] | 1.47 [1.47-1.47] | 0.941 |
| **RSPV-Post** | 0 | 1.13 [1.00-1.33] | NA | NA |
| **RCar-Post** | 3 | 1.33 [1.17-1.50] | 1.40 [1.37-1.77] | 0.159 |
| **RIPV-Post** | 1 | 1.10 [0.97-1.20] | 1.43 [1.43-1.43] | 0.118 |
| **RIPV-Inf** | 0 | 1.08 [1.00-1.17] | NA | NA |
| **RIPV-Ant** | 0 | 1.13 [1.00-1.27] | NA | NA |
| **RCar-Ant** | 6 | 1.43 [1.22-1.50] | 1.80 [1.67-1.83] | **0.001** |
| **RSPV-Ant** | 3 | 1.23 [1.10-1.33] | 1.43 [1.37-1.83] | **0.032** |

All measurements are shown as median [IQR].
Ant = anterior, Inf = inferior, LCar = left carina, LIPV = left inferior pulmonary vein, LSPV = left superior pulmonary vein, NA = not applicable, Post = posterior, RCar = right carina, RIPV = right inferior pulmonary vein, RSPV = right superior pulmonary vein.
